# Universal and Targeted Policy for Health Equity in the Neoliberal Era; A Response to the Recent Commentaries

**DOI:** 10.34172/ijhpm.2023.7998

**Published:** 2023-03-13

**Authors:** Matthew Fisher, Patrick Harris, Toby Freeman, Emma George, Fran Baum

**Affiliations:** ^1^Stretton Health Equity, University of Adelaide, Adelaide, SA, Australia; ^2^Centre for Health Equity Training, Research and Evaluation, University of New South Wales, Sydney, NSW, Australia; ^3^School of Allied Health Science and Practice, University of Adelaide, Adelaide, SA, Australia

 Growing socioeconomic inequalities globally^1^ are likely to exacerbate health inequities within and between countries.^2^ National governments can influence social determinants of health within their own borders, in order to promote health and reduce inequities. Questions about how to do this effectively include debate on use of universal or targeted policies to improve equitable access to health care and other determinants.^3-5^ In 2021 we published research in this journal^6^ examining the merits of universal and targeted policies for health equity, drawing on case studies of Australian policy in primary health care, Indigenous health, telecommunications, and land use planning.^7^ We argued that, compared with market mechanisms, universal policies continue to have an essential role for equity. However, targeted policies also have a role to supplement universal systems and respond to the rights claims or needs of population groups subjected to systemic discrimination or disadvantage. We also found that a network of primary healthcare organisations had some potential for proportionate universalism (PU), allocating resources proportionate to assessed regional needs, within a universalist system. In all cases, specifics about the way a policy is designed, implemented, and governed affected equity of access to health determinants, across dimensions of availability, affordability, and acceptability.^6^ We argued that devolved governance structures had significant potential to enhance equity of outcomes of both universal and targeted strategies, enabling flexible responses to differing needs in different locales.

 In 2022, the journal published commentaries on our article, from Mead et al,^8^ and from Raphael and Bryant.^9^ Their comments were thoughtful and considered and here we reflect on some key points raised. We welcome Raphael and Bryant’s emphasis on the question of *why* government agencies might be reluctant to support devolved governance. They argue that neoliberal political ideology – such as prevails in Australia – has permeated public agencies, resulting in managerial models such as new public management, characterised by top-down control and prescriptive regulation for ‘performance management.’ These trends ‘make achieving the goal of devolving governance processes for promoting equity more difficult.’^9^ We can only agree, and elsewhere we have endorsed the importance of understanding ways in which ideology and power distribution constrain public policy.^10^ However, our work on ‘Closing the Gap’ policy indicates Indigenous communities and representative bodies in Australia have had some success in challenging top-down approaches to policy governance^11^; reinforcing Raphael and Bryant’s further points about resistance.

 Raphael and Bryant’s arguments also call attention to other literature on ‘governance’ examining questions of multiple parties taking a role in controlling public policy, or of ‘multi-level’ governance mechanisms operating across national, regional, or local scales.^12^ Such concepts and practices of governance may favour private sector actors and take little or no account of equity. Indeed, this is what we showed in our study of land-use policy practice surrounding the ‘Western Sydney City Deal’ (WSCD).^7^ There, we found the dimensions of macro-level neoliberal governance identified by Raphael and Bryant did indeed play out, with investment of capital to benefit Western Sydney as the driving objective of the WSCD. Equity, risk, or disadvantage were never on the table. The governance behind the WSCD was centred around government agencies coming together, but to foster investment rather than address equity.^7^ More recently, Harris^13^ has extended that work to show how power and governance intertwine dynamically to structure out pro-equity action in urban policy. Essentially, neoliberal goals in urban investment initiatives like the WSCD lead to governance regimes that perpetuate those goals and ignore ‘risks’ of inequity, climate change or COVID-19. The pro-investment, pro-growth urban governance agenda, Harris demonstrates, dismisses equity as ‘anticapitalistic,’ ‘needy,’ and unappealing for ‘investment.’^14^ Health equity advocates should aim to insert themselves into relevant governance regimes along with pro-equity goals and objectives for accountability.^13^

 In our article, we concluded that a mixed approach involving universal, proportionate, and targeted structures, and devolved governance, will be ‘best suited to achieve equity in affordability, availability and acceptability.’^6^ Mead et al argue that such a conclusion may overlook both the social ‘cohesion dividend’ of universalism and potential stigma associated with targeted policies. Further, they claim that PU may result in practice in targeting resources to areas of low average socioeconomic status, overlooking disadvantage in other areas. They propose an additional equity approach, ‘Equity Sensitive Universalism’ which focuses on ‘achieving proportionate outcomes with equally provided resources.’^8^ We agree that PU is not always the right approach to equitable universalism and requires mechanisms to match resources to need across all areas. However, our example of primary health networks offers just such a mechanism, despite being significantly constrained by limited funding. The example of Equity Sensitive Universalism put forward by Mead et al, a universal child benefit, has been shown to have been equitable in the United Kingdom, noting that it was coupled with progressive taxation to ‘draw back’ funds from affluent parents. Certainly, stigma has been an issue for Indigenous health in Australia in particular, with Aboriginal and Torres Strait Islander peoples taking active steps to oppose colonial deficit-focused representations of their people.^15^ However, when targeted approaches have been community controlled, this can have the opposite effect, boosting cultural identity, pride, and connection.^16^ Linking our original article and both commentaries is a firm conclusion that neoliberalism in Australia, in Canada, and in the United Kingdom, has exacerbated health inequities, and policy approaches that centre equity are urgently needed to displace the current neoliberal approach. The discussion in these rich commentaries around different options to achieve equity through policy can only strengthen potential future policy development.

## Ethical issues

 Not applicable.

## Competing interests

 Authors declare that they have no competing interests.

## Authors’ contributions

 MF wrote the draft correspondence. PH, TF, EG, and FB read and provided feedback on the draft correspondence.
